# Different Regulation of Interleukin-1 Production and Activity in Monocytes and Macrophages: Innate Memory as an Endogenous Mechanism of IL-1 Inhibition

**DOI:** 10.3389/fphar.2017.00335

**Published:** 2017-06-08

**Authors:** Mariusz P. Madej, Elfi Töpfer, Diana Boraschi, Paola Italiani

**Affiliations:** Institute of Protein Biochemistry, National Research CouncilNaples, Italy

**Keywords:** cytokines, inflammation, innate memory, interleukin-1, monocytes, macrophages

## Abstract

Production and activity of interleukin (IL)-1β are kept under strict control in our body, because of its powerful inflammation-promoting capacity. Control of IL-1β production and activity allows IL-1 to exert its defensive activities without causing extensive tissue damage. Monocytes are the major producers of IL-1β during inflammation, but they are also able to produce significant amounts of IL-1 inhibitors such as IL-1Ra and the soluble form of the decoy receptor IL-1R2, in an auto-regulatory feedback loop. Here, we investigated how innate immune memory could modulate production and activity of IL-1β by human primary monocytes and monocyte-derived tissue-like/deactivated macrophages *in vitro*. Cells were exposed to Gram-negative (*Escherichia coli*) and Gram-positive (*Lactobacillus acidophilus*) bacteria for 24 h, then allowed to rest, and then re-challenged with the same stimuli. The presence of biologically active IL-1β in cell supernatants was calculated as the ratio between free IL-1β (i.e., the cytokine that is not bound/inhibited by sIL-1R2) and its receptor antagonist IL-1Ra. As expected, we observed that the responsiveness of tissue-like/deactivated macrophages to bacterial stimuli was lower than that of monocytes. After resting and re-stimulation, a memory effect was evident for the production of inflammatory cytokines, whereas production of alarm signals (chemokines) was minimally affected. We observed a high variability in the innate memory response among individual donors. This is expected since innate memory largely depends on the previous history of exposure or infections, which is different in different subjects. Overall, innate memory appeared to limit the amount of active IL-1β produced by macrophages in response to a bacterial challenge, while enhancing the responsiveness of monocytes. The functional re-programming of mononuclear phagocytes through modulation of innate memory may provide innovative approaches in the management of inflammatory diseases, as well as in the design of new immunization strategies. In this respect, the interindividual variability in innate memory suggests the need of a personalized assessment.

## Introduction

During the last several years, our knowledge on the interleukin (IL)-1 family molecules, as central mediators of innate immunity/inflammation and as “guilty” molecules of the development of autoinflammatory, autoimmune, infectious and degenerative diseases, has increased (Sims and Smith, 2010; Dinarello, 2011a,b, 2013; Dinarello and van der Meer, 2013; Garlanda et al., 2013a) The IL-1 family encompasses 11 cytokines/ligands and 10 related receptors (Dinarello et al., 2010; Boraschi and Tagliabue, 2013). Among the IL-1 family ligands, IL-1β is produced by mononuclear phagocytes in response to infectious or other stressful events, and initiates a potent defensive inflammatory response, while the structurally similar IL-1α is released only upon cell death and functions as an alarmin (Dinarello, 2011a; Rider et al., 2013). The IL-1-induced inflammation is regulated by a complex interaction of receptors and soluble inhibitors, whose concerted action determines the timing of activity and its shut-off. Both IL-1α and IL-1β bind to IL-1R1 and form an activating complex with the signaling chain IL-1R3. The receptor antagonist IL-1Ra binds to IL-1R1 receptor with high affinity, thereby competing with IL-1α and IL-1β, and does not recruit IL-1R3 (thus the complex is inactive). The other IL-1-binding receptor, the decoy receptor IL-1R2, can bind IL-1β and less efficiently IL-1α and IL-1Ra, and can recruit IL-1R3. However, the complex is inactive due to the lack of signal-initiating sequences in the intracellular domain of IL-1R2. The soluble forms of these receptors (sIL-1R1, sIL-1R2, sIL-1R3) have an inhibitory function by acting as ligand traps, and ensure a balance between amplification/activation of defensive responses and uncontrolled inflammation (Boraschi and Tagliabue, 2013; Garlanda et al., 2013b).

IL-1β is primarily produced by hematopoietic cells in response to various microbial stimuli, activated complement components, other inflammatory cytokines (e.g., TNF-α) and IL-1 itself. IL-1β is synthesized as a long inactive pro-form, which needs cleavage within the context of the inflammasome for being activated and then secreted (by non-conventional mechanisms) (Burns et al., 2003; Martinon et al., 2009; Monteleone et al., 2015). Activation of the inflammasome and of the IL-1 cleaving enzyme caspase-1 is therefore an additional mechanism controlling IL-1β-induced inflammation.

IL-1β production may vary, in innate immune cells, depending on the activation status of such cells. The concept of innate immune memory, i.e., the variation of innate reactivity in cells previously exposed to various stimuli, is a concept well known in invertebrates and also in vertebrates, which has been recently re-confirmed in higher vertebrates and humans (Kleinnijenhuis et al., 2012; Netea et al., 2016). A re-programming of innate immune cells can lead to decreased (tolerance) or enhanced (training) reactivity against reinfection by the same or different pathogens. Tolerance aims to avoid extensive tissue damage, whereas training aims to improve tissue surveillance, necessary to protect weakened tissues (Ifrim et al., 2014; Töpfer et al., 2015). It has been known for several decades that priming of mononuclear phagocytes with lipopolysaccharide (LPS) inhibits cellular functions in a process called LPS-induced tolerance (Dobrovolskaia and Vogel, 2002; Fan and Cook, 2004), whereas only recently it has been shown that priming with *Candida albicans* or the fungal cell wall component β-glucan can induce enhanced responses (Quintin et al., 2012). The effect of different types of bacteria (Gram-negative *vs*. Gram-positive) on the development of innate immune memory still remains unclear. While Gram-negative bacterial components such as LPS can lead to innate tolerance and a decreased response, exposure to Gram-positive Bacillus Calmette–Guérin leads to trained immunity and a more effective host immune response, accompanied by a reduced mortality to non-related infections (Quintin et al., 2014; Blok et al., 2015).

A series of recent *in vitro* and *in vivo* experiments has shown that pathogen-associated molecular patterns and a number of danger-associated molecular patterns induce innate immune memory (Netea et al., 2011; Crisan et al., 2016). Molecular and cellular mechanisms involved in this phenomenon are still not fully understood. It is believed that the main mechanism underlying enhanced responses involves epigenetic reprogramming, which in turn may entail altered pattern recognition receptors’ expression, metabolic reprogramming, or/and altered cytokines release (Saeed et al., 2014; Bekkering et al., 2016b). Long-term epigenetic changes in monocytes and macrophages apparently involve both histone methylation and acetylation, as for instance H3K4 monomethylation and H3K27 acetylation induced by LPS (Ostuni et al., 2013; Saeed et al., 2014), and histone H3K4 trimethylation and H3K27 acetylation caused by β-glucan (Quintin et al., 2012; Saeed et al., 2014). Recently, it has been hypothesized that innate memory could also involve the modulation of expression of “latent and *de novo*” enhancers, microRNAs and/or long non-coding RNAs (Netea et al., 2016).

Unraveling the mechanisms at the basis of innate memory could lead to a better understanding of innate host defense and development of new immunization strategies and immunotherapies (Töpfer et al., 2015).

The aim of the present study was to investigate how innate memory can change the inflammatory reactivity of human monocytes and macrophages. Our study particularly focuses on changes in the levels of active available IL-1β produced by cells exposed and re-stimulated with Gram-positive and Gram-negative bacteria and bacterial components. This could set the stage for understanding how innate memory could be exploited for regulating IL-1 family ligands and receptors for innovative therapies of IL-1-mediated diseases, as well as new immunization strategies.

## Materials and Methods

### Human Monocyte Isolation and Macrophage Differentiation

Human monocytes were isolated from fresh buffy coats of healthy donors recruited at the Blood Transfusion Center of Policlinico Hospital in Napoli. The national legislation does not require informed consent or ethical approval for the use of the anonymous, discarded buffy coats. Peripheral blood mononuclear cells (PBMC) were obtained by density gradient centrifugation on Ficoll-Hypaque (GE Healthcare, Bio-Sciences AB, Uppsala, Sweden). Monocytes were isolated from PBMC by positive selection with human CD14 MicroBeads (Miltenyi Biotec, Bergisch Gladbach, Germany). Monocytes (>95% pure) were cultured in RPMI-1640 medium (GIBCO, Life Technologies, Paisley, United Kingdom) containing 5% heat-inactivated human AB serum (Lonza, Walkersville, MD, United States) and 50 μg/ml gentamicin (Sigma-Aldrich, Inc., St. Louis, MO, United States) (culture medium). Cells were cultured at a density of 0.75 × 10^6^ cells/ml/well in 12-well culture plates (Costar, Corning Inc., Corning, NY, United States) at 37°C in humid air with 5% CO_2_. Monocyte stimulation was performed after overnight resting.

The average percentage of CD14^++^CD16^-^ (85.4%), CD14^++^CD16^+^ (2.8%), and CD14^dim^CD16^+^ (7.7%) monocyte subsets after purification by magnetic sorting fully reflected the percentage of the same subpopulations found in PBMC (78.1, 5.5, and 9.0%, respectively). Thus, the monocyte population used in our experiments was representative of the monocyte heterogeneity as present in the circulation and was similar for all the donors.

Freshly isolated monocytes were differentiated into tissue-like macrophages following a previously published protocol (Mia et al., 2014) with slight modifications. Monocytes were cultured in culture medium containing 50 ng/ml macrophage colony-stimulating factor (M-CSF) for 6 days (with one medium change on the third day). After 6 days, cells were additionally exposed for 24 h to M-CSF (50 ng/ml), IL-10 (20 ng/ml), and TGF-β (10 ng/ml), to generate tissue-like/deactivated macrophages (we will refer to these cells as “macrophages” throughout the text).

We have used IL-10 and TGF-β *in vitro* for reproducing the tissue microenvironment in which gut macrophages develop and reside. Gut resident macrophages contribute to maintaining tissue homeostasis by producing robust amounts of IL-10 (Saraiva and O’Garra, 2010; Maheshwari et al., 2011; Bain and Mowat, 2014; Kamada and Núñez, 2014). IL-10 also promotes the differentiation and maintenance of regulatory T (Treg) cells along with TGF-β produced by the intestinal epithelium upon contact with commensal bacteria. Similar conditions are present in other mucosal districts, and for this reason we generically define these *in vitro* differentiated macrophages as “tissue-like.” The concentrations of IL-10 and TGF-β used were selected from dose–response experiments.

All cytokines and factors were obtained from R&D Systems (R&D Systems, Minneapolis, MN, United States). Macrophages were exposed to stimuli immediately after the 7-day differentiation process.

### Phenotypical Characterization of Mononuclear Phagocytes

Freshly isolated human monocytes and *in vitro* generated macrophages were labeled with fluorescein isothiocyanate (FITC)-conjugated anti-CD14, phycoerythrin (PE)-conjugated anti-CD64, anti-CD80, anti-CD206 (BD Biosciences, San Jose, CA, United States) and FITC-conjugated anti-CX3CR1 (BioLegend, San Diego, CA, United States). Monocytes were incubated with antibodies for 30 min at room temperature (RT), then fixed with 4% paraformaldehyde for 15 min at RT, and kept overnight in 4°C prior to analysis. Macrophages were harvested using Macrophage Detachment Solution DXF (PromoCell GmbH, Heidelberg, Germany), stained with antibodies for 30 min at RT and analyzed immediately. Appropriate isotype controls were used as negative controls. Samples were acquired with the BD FACSCanto^TM^ II system (BD Biosciences). Prior to analysis, monocytes and macrophages were gated based on size forward scatter (FSC) and granularity side scatter (SSC) in order to eliminate other cell types, dead cells, or debris. Analysis was performed using the FlowJo v10.0.7 software (Tree Star, Inc., Ashland, OR, United States). Monocytes were CD14^+^ CD64^+^ CX3CR1^+^ CD206^-^ CD80^-^, whereas macrophages were heterogeneous, with about 50% expressing CD14 and CD64, whereas in general they were positive for CX3CR1 and CD206, and negative for CD80.

### *In Vitro* Stimulation of Monocytes and Macrophages

Monocytes and macrophages were exposed to different bacteria at a bacteria:cell ratio of 10:1. The bacteria:cell ratio was chosen from dose–response experiments. Bacteria used were heat-inactivated Gram-negative *Escherichia coli* (strain BL12-pLysE) and commensal Gram-positive *Lactobacillus acidophilus* (strain LPLANE20174). As positive control, LPS (10 ng/ml) from *E. coli* serotype O55:B5 (Sigma-Aldrich, Inc.) was used. Supernatants were collected after 24 h, centrifuged at 500 × *g* for 5 min and stored at -80°C until analysis.

### *In Vitro* Model of Innate Memory

Monocytes and macrophages were incubated with a low dose of LPS (1 ng/ml) or a low ratio of *E. coli* or *L. acidophilus* to monocytes/macrophages (0.1:1) for 24 h (priming), then supernatants were collected and cells maintained in culture medium for 6 additional days. Medium was changed after 3 days for monocytes and every second day for macrophages. After the resting period, supernatants were collected and cells were challenged with a higher dose of the same stimulus (10 ng/ml LPS, bacteria at a ratio of 10:1) for 24 h. Controls included unprimed cells (exposed only to the challenge) unchallenged cells (exposed only to the priming) and unprimed/unchallenged cells. Supernatants were centrifuged at 500 × *g* for 5 min and stored at -80°C until analysis.

### Cytokine Measurements

Levels of IL-1β, IL-1Ra, TNF-α, IL-8/CXCL8, and MCP-1/CCL2 were measured in monocyte and macrophage supernatants by ELISA (R&D Systems). The IL-1 family cytokines (IL-1α, IL-1β, IL-18, IL-33), and receptors and accessory proteins (sIL-1R1, sIL-1R2, sIL-1R3, sIL-1R4, IL-18BP) were measured using a multiplex assay technology and software custom-developed by Quansys Biosciences, Inc. (Logan, UT, United States). All measurements and analyses were performed according to the manufacturer’s instructions. IL-1α, IL-18, IL-33, sIL-1R4, and IL-18BP were minimally produced (data are not shown in Section “Results”).

### Assessment of Free and Active IL-1β

The calculation of free IL-1β in cell culture supernatants was done by applying the law of mass action, similarly to the calculation of free IL-18 (Novick et al., 2001; Migliorini et al., 2010). The calculation considers that in culture supernatants the levels of sIL-1R1 and sIL-1R3 are stable (data not shown) and that the concentrations of IL-1α are minimal. In these circumstances, the major soluble ligand of IL-1β is sIL-1R2, a molecule that has good affinity for IL-1β (2.7 nM) but low affinity for IL-1Ra (25 μM).

The law of mass action was therefore rearranged to account for the free ligand concentration ([L_F_], see below) according to Clark’s theory (i.e., one ligand, one receptor, specific binding).

$$\left[L_{F}\right]=\frac{-[R_{T}]+[L_{T}]-K_{d}+\sqrt{{\left([R_{T}]-[L_{T}]+K_{d}\right)}^{2}+4[L_{T}]\times K_{d}}}{2}.$$

Where:

R_T_: concentration of sIL-1R2 in pg/ml (MW 47 kDa).

L_T_: concentration of IL-1β in pg/ml (MW 17 kDa).

K_d_: dissociation constant (2.7 nM; Symons et al., 1995).

Active IL-1β was calculated as a ratio between free IL-1β (calculated as above) and IL-1Ra, multiplied by 1000.

Active IL-1β = (free IL-1β/IL-1Ra) × 1000.

### Statistical Analysis

All values were expressed as mean ± SD. Mann–Whitney *U*–test or independent samples *t*-test were employed to compare results from different treatments. Statistics was analyzed using the GraphPad Prism 6 software. Values of *P* < 0.05 were considered statistically significant.

## Results

### Reactivity of Human Monocytes and Macrophages to Gram-Positive and Gram-Negative Bacteria

**FIGURE 1:**
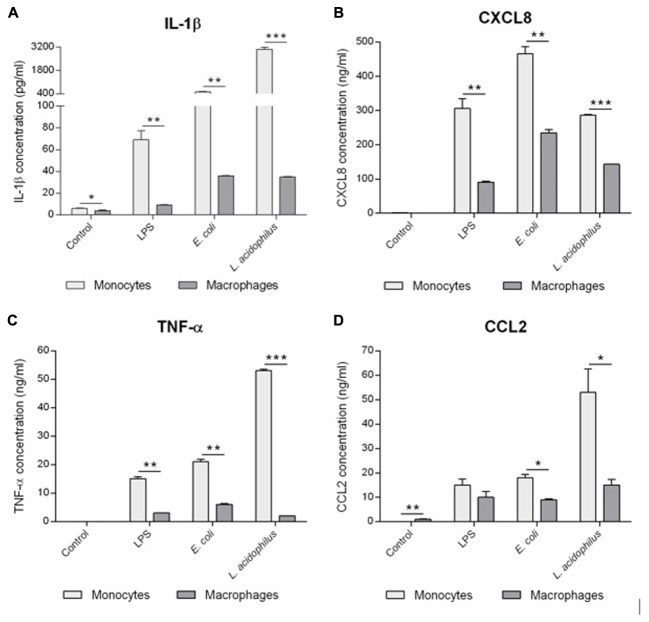
Reactivity of human monocytes and macrophages to bacteria. Human monocytes and monocyte-derived macrophages were cultured in the absence or presence of LPS (10 ng/ml), *E. coli* or *L. acidophilus* (bacteria:cell ratio 10:1) for 24 h. The production of IL-1β **(A)**, CXCL8 **(B)**, TNF-α **(C)**, and CCL2 **(D)** was determined in the supernatant by ELISA. Data from one representative donor. Values shown are means of two independent determinations. The independent samples *t*-test was used to detect significant difference. ^∗^*P* < 0.05, ^∗∗^*P* < 0.01, and ^∗∗∗^*P* < 0.001. The difference between controls and treatments are all statistically significant. The *P*-value is not indicated to avoid overwriting the figure.

The response of human monocytes and macrophages to stimulation with bacteria *in vitro* was measured in terms of production of inflammatory cytokines (IL-1β, TNF-α) and chemokines (CXCL8, CCL2). As shown in **Figure 1**, monocytes produced higher amounts of cytokines compared to macrophages from the same donor after exposure to bacterial LPS, and whole bacteria *E. coli*, and *L. acidophilus*. More specifically, response to LPS was 4.1- and 7.3-fold higher in terms of TNF-α and IL-1β production, respectively, while responses to *E. coli* were 3.5- and 14.1-fold higher, and those to *L. acidophilus* were 21.1- and 86.0-fold higher. Thus, macrophages produce much less IL-1β than monocytes, whereas the difference in terms of TNF-α production is less evident. Even less evident is the difference in chemokine production, with monocytes producing 3.5- and 1.4-fold more CXCL8 and CCL2 in response to LPS, 2.0-fold more in response to *E. coli*, and 2.0- and 3.5-fold more in response to *L. acidophilus*. Thus, while overall the responsiveness of tissue-like/deactivated macrophages to bacterial stimuli is limited, when compared to monocytes, it is obvious that the capacity of producing alarm signals such as chemokines is less affected than the ability to produce inflammatory/destructive factors.

### Innate Memory Is Not Stimulus-Specific

**FIGURE 2:**
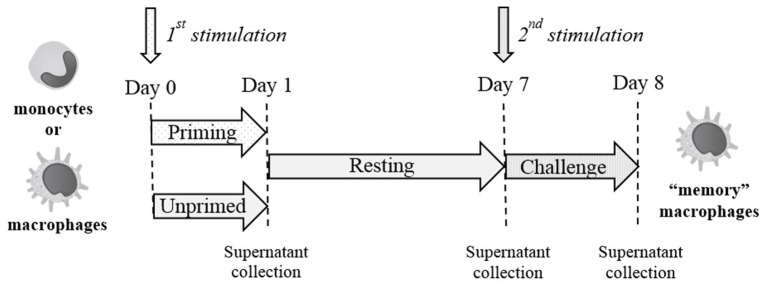
Schematic representation of the *in vitro* model for generating innate memory.

**FIGURE 3:**
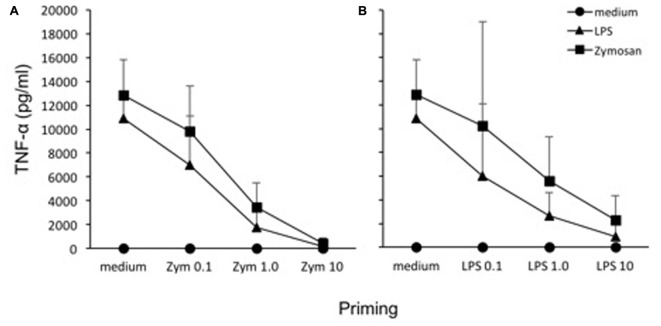
Lack of specificity of innate memory. Monocytes were primed with different doses of Zymosan (0.1, 1, 10 μg/ml) **(A)** or LPS (0.1, 1, 10 ng/ml) **(B)** for 24 h, rested for 6 days, and challenged for 24 h with high doses (10 ng/ml LPS, 10 μg/ml Zymosan) of the same or different stimulus. Production of TNF-α was measured by ELISA. The values were expressed as average ± SD of *n* = 3 donors. The Mann–Whitney *U*-test was used to detect significant difference. The difference between controls and treatments are all statistically significant (*P* < 0.001), except at the highest priming concentration.

An *in vitro* model based on human primary mononuclear phagocytes was set up, in order to study the development of innate memory upon microbial stimulation. Priming was performed by exposing cells to a low concentration of the stimulus that was later used at higher concentrations as challenge (**Figure 2**). To confirm the notion that innate memory is not stimulus-specific (i.e., that the priming stimulus does not need to be the same as the challenge agent) we have performed preliminary experiments by assessing the generation of innate memory *in vitro* upon homologous *vs*. cross-stimulation. In the representative experiment shown in **Figure 3**, the generation of memory was assessed by measuring the production of TNF-α by monocytes primed with either one of two microbial agents (bacterial LPS, yeast Zymosan) in response to challenge with the same or with the other agent. Data in **Figure 3** show that, compared to unprimed monocytes, primed cells respond to challenge with either agent with a decrease of TNF-α production. Such decrease depends on the dose of the priming agent but it is independent of its nature. Thus, priming with LPS induced TNF-α decrease in response not only to the homologous LPS challenge but also to the unrelated challenge with Zymosan, and *vice versa*. Having confirmed the notion of lack of specificity in innate memory, in this study we have performed the memory experiments using the same stimulus for both priming and challenge.

### Development of Innate Immune Memory: Re-programming of Inflammatory Reactivity

**FIGURE 4:**
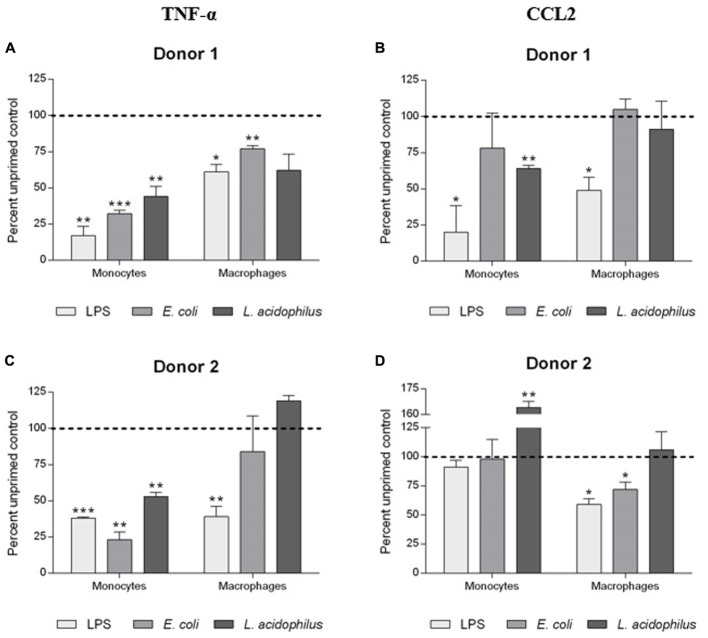
Development of innate memory in monocytes and macrophages. Monocytes and monocyte-derived macrophages were primed with low doses of LPS (1 ng/ml), *E. coli* (0.1:1), or *L. acidophilus* (0.1:1) for 24 h, rested for 6 days, and challenged for 24 h with high doses of the same stimuli (10 ng/ml LPS, *E. coli* and *L. acidophilus* at 10:1). Production of TNF-α **(A,C)**, and CCL2 **(B,D)** was measured by ELISA. Data are presented as percentage of the cytokine amount produced by unprimed cells (dotted line). Representative data from two different donors are shown. Values shown are means of two independent determinations for each donor. Absolute mean TNF-α values upon stimulation with LPS were 1.56 and 2.56 ng/ml for monocytes of donors 1 and 2, respectively; and 1.15 and 20.92 ng/ml for macrophages of the two donors. Absolute mean CCL2 values were 14.91 and 11.17 ng/ml for monocytes of donors 1 and 2, while for macrophages values were 14.95 and 7.91 ng/ml. The independent samples *t*-test was used to analyze statistically significant differences. ^∗^*P* < 0.05, ^∗∗^*P* < 0.01, and ^∗∗∗^*P* < 0.001.

The generation of memory was assessed by measuring the production of TNF-α and CCL2 by primed cells in comparison to the levels of cytokines produced by unprimed cells using the same stimulus for priming and challenge (i.e., LPS, *E. coli*, and *L. acidophilus*; **Figure 4**).

Primed monocytes secreted less than half the amount of TNF-α compared to unprimed cells, independently of the stimulus (LPS or whole bacteria). In the case of CCL2, the donor-to-donor variation in the monocyte response was evident, with a decrease of production observed in response to LPS (donor 1), and little/no variation (bacteria in donor 1, LPS and *E. coli* in donor 2), or a significant increase (*L. acidophilus* in donor 2) in other cases. The situation is different in macrophages, in which the decrease in cytokine production due to memory is much less evident, and in general clear only in the case of LPS and practically absent with *L. acidophilus*.

### Innate Memory Can Modulate IL-1 Production

We have examined more in detail the effect of innate memory on the production of cytokines of the IL-1 family by monocytes and macrophages. Here we mainly focus on the IL-1 system, i.e., the agonist cytokines IL-1α and IL-1β, their receptor antagonist IL-1Ra, and the soluble receptors sIL-1R1, sIL-1R2, and sIL-1R3. IL-1α was minimally produced in our culture conditions (data not shown). Indeed this cytokine may be detected in cell culture supernatants of stressed cells that undergo necrosis or pyroptosis (Dinarello, 2013). IL-1β is also minimally produced by unstimulated mononuclear phagocytes (data not shown). On the other hand, macrophages constitutively produced around 35-fold higher amounts of IL-1Ra than monocytes (data not shown). Regarding the soluble receptors, monocytes released amounts of sIL-1R1 and sIL-1R3 comparable to those of macrophages, whereas macrophages released more sIL-1R2 than monocytes (**Table 1** and data not shown). Priming of monocytes or macrophages with low doses of stimuli did not significantly affect the release of soluble IL-1R1 and IL-1R2 after challenge (**Table 1**). A lack of memory was also observed for sIL-1R3, although its levels were significantly variable between donors (data not shown).

**Table 1:** Production of sIL-1R1 and sIL-1R2 by monocytes and macrophages upon challenge with bacterial stimuli.

Stimulus	sIL-1R1 (pg/ml)		sIL-1R2 (pg/ml)	
	Unprimed	Primed	Unprimed	Primed
**Monocytes**				
medium	63.1 ± 2.6		156.1 ± 39.0	
LPS	53.9 ± 16.5	47.9 ± 8.3	155.7 ± 77.4	130.8 ± 83.2
*E. coli*	56.1 ± 23.5	48.1 ± 19.9	144.7 ± 51.9	174.3 ± 56.9
*L. acidophilus*	51.6 ± 13.8	53.5 ± 19.7	136.3 ± 49.6	196.3 ± 33.5
**Macrophages**				
medium	44.9 ± 14.6		1409.9 ± 952.3	
LPS	61.9 ± 9.1	54.7 ± 11.4	1101.6 ± 482.5	596.9 ± 192.6
*E. coli*	58.5 ± 14.9	57.1 ± 18.1	998.7 ± 191.9	1041.4 ± 131.9
*L. acidophilus*	53.4 ± 12.1	58.7 ± 9.1	1092.9 ± 544.9	1126.9 ± 630.4

*The values were expressed as average ± SD of *n* = 3 donors. Two independent determinations for each donor were performed. No statistically significant differences were detected with Mann–Whitney *U*-test*.

**FIGURE 5:**
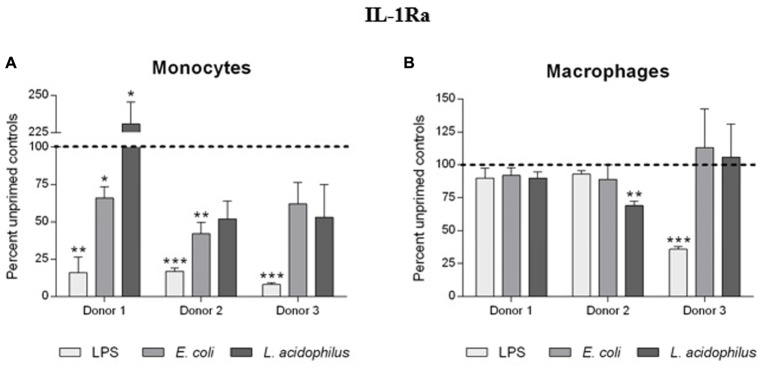
Influence of innate memory on the production of IL-1Ra by monocytes and macrophages. Monocytes and monocyte-derived macrophages were primed with low doses of LPS (1 ng/ml), *E. coli* (0.1:1) or *L. acidophilus* (0.1:1) for 24 h, rested for 6 days, and challenged for 24 h with high doses of the same stimuli (10 ng/ml LPS, *E. coli* and *L. acidophilus* 10:1). Production of IL-1Ra by monocytes **(A)** and macrophages **(B)** was measured by ELISA. Data are presented as percentage of the cytokine amount produced by unprimed cells (dotted line). Representative data from three different donors are shown. Values shown are means of two independent determinations for each donor. Absolute mean values of IL-1Ra production upon LPS stimulation were 43.73, 36.78, and 14.21 ng/ml for monocytes of donors 1, 2, and 3, respectively; IL-1Ra produced by macrophages of the same donors in response to LPS was 66.62, 146.67, and 43.99 ng/ml. The independent samples *t*-test was used to analyze statistically significant differences. ^∗^*P* < 0.05, ^∗∗^*P* < 0.01, and ^∗∗∗^*P* < 0.001.

Production of IL-1Ra was strongly affected by priming of monocytes with bacterial stimuli (**Figure 5**). In monocytes primed with LPS, a challenge with a higher LPS concentration resulted in 80–90% inhibition of IL-1Ra production as compared to unprimed cells. The “tolerance” was evident also with *E. coli* as stimulus, whereas with *L. acidophilus* there was variability among donors (with 1/3 actually showing increased IL-1Ra production). In contrast, no substantial changes in IL-1Ra production were observed in macrophages, except for pre-treatment with LPS for one donor (**Figure 5**).

**FIGURE 6:**
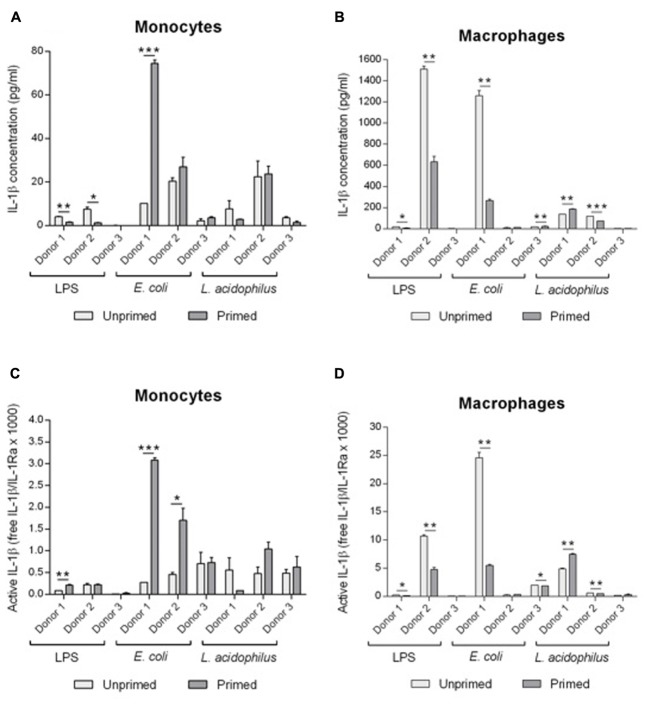
Modulation of active IL-1β levels by innate memory. Monocytes and monocyte-derived macrophages were primed with low doses of LPS (1 ng/ml), *E. coli* (0.1:1) or *L. acidophilus* (0.1:1) for 24 h, then rested for 6 days, and challenged for 24 h with high doses of the same stimuli (10 ng/ml LPS, *E. coli* and *L. acidophilus* 10:1). The concentrations of IL-1β **(A,B)** were: for donor 1, unprimed monocytes have a value of about 0.28 pg/ml and macrophages 24.59 pg/ml; for donor 2 monocytes have 0.46 pg/ml and macrophages 0.23 pg/ml; for donor 3 monocytes have 0.71 pg/ml and macrophages 2.39 pg/ml. Free IL-1β was calculated by applying the law of mass action, and active IL-1β **(C,D)** was determined as a ratio between free IL-1β and its antagonist IL-1Ra. Representative data from three different donors are shown. Values shown are means of two independent determinations for each donor. The independent samples *t*-test was used to detect significant difference. ^∗^*P* < 0.05, ^∗∗^*P* < 0.01, and ^∗∗∗^*P* < 0.001.

To better evaluate the functional significance of modulation of IL-1 family cytokines and receptors consequent to memory generation, we have calculated the actual presence of free biologically active IL-1β. First, we have measured by ELISA the production of immunoreactive IL-1β produced by monocytes and macrophages (**Figures 6A,B**), and in the same samples we have measured the concentration of produced sIL-1R2 (data not shown) and of IL-1Ra (**Figure 5**). Free IL-1β was calculated according to the law of mass action, based on a stoichiometric 1:1 ratio between the cytokine and sIL-1R2 and a binding affinity of 2.7 nM. To have a measure of active IL-1β, i.e., the amount of free IL-1 that is not blocked by IL-1Ra, we have used the ratio between free IL-1β and IL-1Ra (**Figures 6C,D**).

Unprimed monocytes produced low levels of active IL-1β (**Figure 6C**). Upon challenge, active IL-1β did not change significantly in monocytes primed with LPS, whereas priming with *E. coli* induced an increase of active IL-1β in 2/3 donors (no change in donor 3). Eventually, memory induced by *L. acidophilus* resulted in variable donor-dependent effects on active IL-1β levels (either decreased or increased production).

Unprimed macrophages produced in general similar or higher levels of active IL-1β than monocytes in response to the various stimuli (**Figure 6D**). Memory induced by LPS caused a significant decrease of active IL-1β. Priming with *E. coli* significantly decreased the levels of active IL-1β in one donor (the one that showed a high reaction to challenge), whereas macrophages from the other donors had a low reactivity that was not significantly changed by priming. Similar to monocytes, memory induced by *L. acidophilus* in macrophages resulted in variable donor-dependent effects on active IL-1β levels. Thus, LPS-induced memory can decrease active IL-1β in macrophages but not in monocytes, while *E. coli*-induced memory increased active IL-1β in monocytes and could decrease it in macrophages, and *L. acidophilus*-induced memory variably affected the levels of active IL-1β in both cell types.

It is noteworthy that when looking at IL-1β (**Figures 6A,B**), LPS-induced tolerance is evident in both monocytes and macrophages of donors 1 and 2 (values of donor 3 were too low). But when the levels of active IL-1β were calculated, i.e., when considering the concomitant production of inhibitors, this decrease is not evident any longer except in one case (macrophages of donor 2). Thus, the control of IL-1β activity is more complex than simple decrease of cytokine production and depends on the concomitant modulation of other regulatory factors.

## Discussion

Regulation of the activity of IL-1β is of paramount importance for obtaining optimal defensive reactions without causing excessive tissue damage. Indeed, the mechanisms of IL-1 regulation are multiple and at all levels, from regulation of its maturation/secretion to regulation of its activity both at the receptor binding level and at the level of post-receptor signaling. This kind of regulation reflects the powerful biological activity of IL-1β that, if not properly controlled, can be at the basis of numerous inflammatory and degenerative diseases (Dinarello, 2011a,b).

In this study, we have investigated a novel mechanism of IL-1β regulation, through the generation of innate memory.

Several studies have recently revived the old concept of innate immune memory, i.e., the ability of innate immune cells to “remember” previous encounters with microorganisms or foreign agents by changing their reactivity to a subsequent challenge with the same or with a different agent (Netea et al., 2011; Saeed et al., 2014; Gardiner and Mills, 2016). The concept, well known in invertebrates and also in mice, applies also to human innate immune cells *in vivo* and *in vitro* (Quintin et al., 2012; Arts et al., 2015; Bekkering et al., 2016a; van der Valk et al., 2016). In our study, we have used an *in vitro* system, based on human primary monocytes and tissue-like/deactivated monocyte-derived macrophages from the same donors, to examine the role of innate memory in determining the production of active IL-1β. We have used as stimuli two bacteria, the Gram-negative *E. coli* and the Gram-positive *L. acidophilus*, and a molecule derived from *E. coli*, i.e., its cell wall LPS. Data presented here were obtained by using the same stimulus for both priming and challenge of cells in culture. However, preliminary data of cross-stimulation confirm the notion that innate memory is not stimulus-specific (**Figure 3**).

The first observation we made is that monocytes are more reactive than macrophages to microbial stimuli in terms of production of inflammatory cytokines and chemokines (**Figure 1**). Although this was expected, it is noteworthy that while the difference in the production of inflammatory cytokines (IL-1β, TNF-α) is significant (up to almost 100×), there is much less difference in the production of chemokines (CXCL8, CCL2). Weaker response of macrophages in terms of inflammatory effector cytokines but almost normal response in terms of alarm signals (such as chemokines) is in line with the sentinel role of tissue-resident macrophages, which should be active in producing chemokines such as CXCL8 and CCL2, aiming at recruiting effector cells (e.g., neutrophils and blood circulating monocytes), but not directly involved in the very early effector phase of inflammation, in order to avoid uncontrolled tissue damage (Davies et al., 2013).

The innate immune memory appears as a decreased (tolerance) or increased response (trained innate immunity) to a second immune challenge. Both tolerance and trained innate immunity seem to be the result of long-term epigenetic changes in monocytes and macrophages, and the ability of these cells to “remember” is actually dependent on epigenetic changes. All the epigenetic changes and molecular mechanisms at the basis of memory eventually result in lower or higher transcription levels in several genes, such as pathogen recognition receptors, signaling molecules, and cytokines, in a short window of time. For example, priming of human monocytes with IFN-γ or IL-10, or the interaction with microbial components, alters the receptor repertoire expressed by monocytes/macrophages, e.g., changes in TLR4 and MD2, and in Dectin-1 and MARCO in macrophages (Bosisio et al., 2002; Willment et al., 2003), or in signaling molecules such as MyD88 (Bosisio et al., 2002). These changes in the number of receptors and the regulation of signaling drive a different production of effector molecules, such as inflammatory and anti-inflammatory cytokines. Although in this study we did not investigate the molecular mechanisms underlying the difference of responsiveness between monocytes and macrophages in terms of cytokine production, it is possible to speculate that the two cell types may undergo different epigenetic changes. In fact, although macrophages in this study are derived from monocytes, the *in vitro* differentiation protocol with deactivating cytokines and growth factors yields a cell population that differs from monocytes also in terms of the level of innate receptors (Ifrim et al., 2014). It is plausible that chromatin modifications occur during the differentiation process, and, when macrophages are primed and challenged, this is translated in a transcription programs different from that of monocytes.

In our *in vitro* model of innate memory, we observed two important facts. First, that also in the case of memory there is little effect on chemokine production, suggesting that the ability of cells to send alarm signals should not be hampered. A second observation is that memory is not induction of tolerance or induction of enhanced responses. In fact, the same stimulus can induce decreased or increased responses in primed cells depending on the endpoint measured (production of one or another of the various inflammation-related factors) and on the cell type examined (monocytes *vs*. macrophages). Thus, innate memory is a complete re-programming of the reactivity of cells rather than a decreased or enhanced responsiveness.

We focused on how innate memory could affect the production of active IL-1β. For doing this we have separately measured the production of IL-1β and of a number of soluble factors, concomitantly produced by monocytes and macrophages, that could affect its biological activity. The inflammatory activity of IL-1β is similar to that of IL-1α, which, however, is a cytokine mostly active intracellularly or as a cell-associated factor in the cell–cell communication (Kim et al., 2013; Di Paolo and Shayakhmetov, 2016; Bertheloot and Latz, 2017). Indeed, we found that IL-1α was practically absent in the supernatants of monocyte and macrophage cultures stimulated with the various stimuli (data not shown). Thus, the IL-1-like activity in these supernatants can be exclusively attributed to IL-1β. It is known that the soluble form of IL-1R2 is a major inhibitor of IL-1β, as it can capture it in solution with high affinity (2.7 nM), while unable to bind with sufficient affinity either IL-1α (1.6 μM) or IL-1Ra (25 μM) (Symons et al., 1995). Thus, sIL-1R2 can be considered as a specific inhibitor of IL-1β. Monocytes and macrophages also produce significant levels of the soluble forms of the other two IL-1 receptor chains, i.e., sIL-1R1 and sIL-1R3. The IL-1-binding sIL-1R1 possibly binds IL-1Ra with higher affinity than IL-1α and IL-1β (Arend et al., 1994), while sIL-1R3 might form complexes with sIL-1R2 of higher affinity for IL-1α and IL-1β but not IL-1Ra (Smith et al., 2003). In these circumstances, since the levels of both sIL-1R1 and sIL-1R3 were constant in monocyte and macrophage supernatants and did not depend on stimulation or memory (**Table 1** and data not shown), we decided to exclude them from the calculation of IL-1β activity. Thus, we have limited the calculation to two elements, IL-1β and sIL-1R2, binding to each other at a 1:1 stoichiometric ratio and with a known affinity (2.7 nM). It was therefore possible to apply the law of mass action to such interaction and to calculate the amount of free IL-1β, i.e., the amount of cytokine not blocked by sIL-1R2 and therefore available for activating target cells by binding to membrane receptors. However, since the presence of IL-1Ra is expected to compete with free IL-1β for receptor binding, we have also taken into account its presence, in order to have a more thorough evaluation of biologically active IL-1β. IL-1Ra is abundantly produced by monocytes (0.9–3.0 ng/ml) and more abundantly by macrophages (9–150 ng/ml), is upregulated upon stimulation (2–7×; data not shown) and it varies depending on memory (**Figure 4**). Thus, its presence is relevant for determining the final capacity of IL-1β to exert its inflammatory effects. In the absence of reliable data for accurately calculating possible competition between the two ligands in a complex system such as the membrane of a responding cells (in which activation is regulated not only by the quantitative presence of the receptor chains but also by the changes in affinity due to the presence of accessory chains and inhibitory receptors), we have indicatively expressed the levels of active IL-1β as the ratio between free IL-1β and IL-1Ra. We have observed several very interesting phenomena. The first observation is, as expected, that each individual subject responds differently, both in quantitative terms (the amount of cytokine produced) and also in terms of type of response (enhanced reaction *vs*. decreased response). This behavior most likely depends on the past history of exposure of the donor, i.e., age, vaccinations, diseases, etc. Another important observation is the different behavior of macrophages (the tissue-resident mononuclear phagocytes) as opposed to monocytes (the blood-borne inflammatory cells). As an example, monocytes of donor 1 reacted to challenge with *E. coli* with a sharp increase in active IL-1β, whereas macrophages of the same donor significantly down-regulated active IL-1β production upon challenge with *E. coli*. This underlines the different role of the two cell types in inflammatory reactions, with monocytes being the inflammatory effector cells that must be ready and reactive, whereas tissue macrophages must avoid excessive reaction that would cause unwanted tissue damage. Another observation that warrants attention is the different response raised by whole *E. coli* bacteria as opposed to the bioactive *E. coli* LPS molecule. For instance, in the case of donor 2, active IL-1β produced by unprimed monocytes in response to LPS is lower than that induced by *E. coli* and is not changed by priming, whereas response to *E. coli* is significantly reduced by priming. Macrophages of the same donor are responsive to LPS but practically unresponsive to *E. coli*, and their response is reduced by priming with LPS but not by priming with *E. coli*. Also, it is notable that macrophages seem to respond either to whole *E. coli* or to LPS, but not to both (see donors 1 and 3: no response to LPS *vs*. significant response to *E. coli*; and donor 2: good response to LPS and undetectable response to *E. coli*).

Our conclusions are based on a preliminary assessment on three donors, and the very high inter-individual variability of the findings may lead to over-interpretation of the data. On the other hand, since the reactivity of the immune system is tailored on previous experiences, it is in a way expected that each subject could respond differently, based on his/her individual immunological history. Thus, increasing the number of subjects may not overcome the issue of inter-individual variability. What we would like to underline with these data is the importance of an individual evaluation of the innate memory status, in view of future approaches of precision medicine that could help us improving/optimizing immunotherapeutic strategies. In cases as that of innate memory, it seems clear that no general conclusions can be drawn, and in these circumstances a case-by-case assessment becomes crucial.

Overall, it could be concluded that innate memory limits the amount of active IL-1β produced by tissue macrophages in response to a challenge, in line with the hypothesis that these cells are sentinels and not effector cells, and should avoid damaging the tissue by initiating a potentially destructive inflammatory response. On the other hand, memory tends to enhance the reaction of monocytes, which are the effector cells in inflammatory reactions, thereby making them more efficacious in combating the potential danger. Thus, the induction of innate memory could help increasing the host resistance to infections without causing excessive local tissue damage. This could be among the reasons for the efficacy of adjuvants in enhancing protective immunity, in addition to facilitating the establishment of adaptive memory. It is exciting the hypothesis that manipulation of innate memory may also become an important therapeutic strategy in chronic inflammatory and degenerative diseases.

## Author Contributions

MM performed the experiments and wrote the paper; ET performed cross-stimulation experiments; PI and DB designed the study and wrote the manuscript.

## Conflict of Interest Statement

The authors declare that the research was conducted in the absence of any commercial or financial relationships that could be construed as a potential conflict of interest.
